# Supplementary material for: Assessing the Relationship between the Gut Microbiota and Inflammatory Bowel Disease Therapeutics: A Systematic Review
Source: Pathogens. 2023 Feb 6;12(2):262. doi: 10.3390/pathogens12020262 (PMC9965214; doi:10.3390/pathogens12020262)
Supplement: Supplementary file 1 [file pathogens-12-00262-s001.zip › pathogens-2060850-supplementary.pdf]

Supplementary Table S1. Newcastle-Ottawa Scale—Summary of Newcastle-Ottawa Scale of each included cohort study.

	**Selection**				**Comparability**	**Outcome**			
**Study**	**Representativeness of the exposed cohort**	**Selection of the non-exposed cohort**	**Ascertainment of exposure**	**Demonstration that outcome of interest was not present at start of study**	**Comparability of cohorts on the basis of the design or analysis controlled for confounders**	**Assessment of outcome**	**Was follow-up long enough for outcomes to occur**	**Adequacy of follow-up of cohorts**	**Total quality score**
Zhou et al. [57]	1	1	1	1	2	1	1	1	9
Kowalska-Duplaga et al. [54]	0	1	1	1	2	1	1	1	8
Ribaldone et al. [53]	1	1	0	1	2	1	1	1	8
Kolho et al. [43]	1	1	1	1	2	1	1	0	8
Ananthakrishnan et al. [42]	1	1	1	1	1	1	1	1	8
Wang et al. [39]	1	1	1	1	2	1	1	1	9
Wang et al. [38]	1	1	1	1	2	1	1	1	9
Aden et al. [58]	1	1	1	1	1	1	1	1	8
Zhuang et al. [40]	1	1	1	1	2	1	1	1	9
Ding et al. [48]	0	1	0	1	2	1	1	1	7
Dovrolis et al. [59]	0	1	1	1	2	1	1	1	8
Olbjorn et al. [50]	1	1	1	0	0*	1	1	1	6
Salamon et al. [55]	1	1	1	1	2	1	1	1	9
Hart et al. [35]	1	1	1	1	2	1	1	1	9
Ishikawa et al. [37]	0	1	1	0	2	1	1	1	7
Kaakoush et al. [64]	1	1	1	1	2	1	1	1	9
Quince et al. [65]	1	1	1	1	2	1	1	1	9

Tang et al. [66]	1	1	1	1	1	1	1	8
Dunn et al. [67]	1	1	1	1	2	1	1	1	9
Costa-Santos et al. [68]	1	1	1	1	2	1	1	1	9
Diederen et al. [69]	1	1	1	1	1	1	1	1	8
Valcheva et al. [51]	1	0	1	1	0*	1	1	1	6
Suskind et al. [52]	1	0	1	1	0*	1	1	1	6
Ventin-Holmberg et al. [56]	1	0	1	1	2	1	1	1	8
Schierova et al. [60]	1	1	1	1	2	1	1	1	9
Sanchis-Artero et al. [61]	1	0	1	1	1	1	1	1	7
Park et al. [63]	1	1	1	1	2	1	1	1	9
Ventin-Holmberg et al. [49]	1	0	1	1	2	1	1	1	8
Effenberger et al. [62]	1	1	1	1	2	1	1	1	9
Wilson et al. [72]	1	0	1	1	2	1	1	1	9
Olendzki et al. [74]	1	1	1	1	2	1	1	1	9
Tang et al. [66]	1	1	1	1	2	1	1	1	9
Jiang et al. [70]	1	0	0	1	2	1	1	1	7
Chen et al. [73]	1	1	1	1	1	1	1	1	8

Note: *No study controls or exclusion criteria involving IBD patients were stated in Olbjorn et al.'s or Suskind or Valcheva manuscripts.

Supplementary Table S2. Summary of Newcastle-Ottawa Scale of each included RCT.

	Selection				Comparability	Exposure			
**Study**	**Is the case definition adequate?**	**Representativeness of the cases**	**Selection of Controls**	**Definition of Controls**	**Comparability of cases and controls on the basis of the design or analysis**	**Ascertainment of exposure**	**Same method of ascertainment for cases and controls**	**Non-Response rate**	**Total quality score**
Doherty et al. [41]	1	1	1	1	2	1	1	1	9
Sprockett et al. [36]	1	1	1	1	2	1	1	1	9
Pigneur et al. [34]	1	0	0	1	2	0	1	1	6
Schierova et al. [33]	1	1	0	1	2	0	1	1	7
Suskind et al. [71]	1	1	1	0	1	1	1	1	7
